# Infantile Iatrogenic Cushing Syndrome due to Topical Steroids

**DOI:** 10.1155/2019/2652961

**Published:** 2019-12-02

**Authors:** Lulwah Alkhuder, Horia Mawlawi

**Affiliations:** Department of Pediatric Endocrinology, Prince Sultan Medical City, Riyadh, Saudi Arabia

## Abstract

Cushing syndrome is an endocrinological disorder characterized by increased free plasma glucocorticoids level. It is either due to an excessive endogenous release of steroids (e.g., pituitary adenoma or adrenal hyperplasia) or exogenous administration of steroids. In children, iatrogenic Cushing syndrome is the most common form of Cushing syndrome occurring in this age group. The vast majority of cases are due to oral or parenteral preparation of steroids, which are commonly prescribed for pulmonary, hematological, renal, or autoimmune pathologies. Topical preparations can rarely cause Cushing syndrome in young children, and only a few cases were reported in the literature, where the patients were older than 5 months of age. In this report, we present a three-month-old girl who developed iatrogenic Cushing syndrome due to prolonged and inappropriate use of topical clobetasol cream for napkin dermatitis.

## 1. Introduction

Cushing syndrome, an endocrinological disorder resulting from abnormally high plasma cortisol levels, is characterized by increased free plasma glucocorticoids level [1]. It can result from either endogenous release of excess steroids (e.g., adrenal gland tumors, adrenal hyperplasia, or pituitary adenoma) or exogenous use of corticosteroid medications such as iatrogenic Cushing syndrome [1]. Clinically, patients with Cushing syndrome present with facial plethora and edema, giving the appearance of “moon face”; accumulation of fat in the supraclavicular area and upper trunk, giving the appearance of “buffalo hump;” accumulation of fat around the waist leading to truncal obesity, which leads to skin fragility resulting in appearance of purple stria, particularly on the abdomen, arms, and upper thighs. Furthermore, it causes hirsutism, skin bruises, ecchymoses, delayed wound healing, proximal muscle wasting, arterial hypertension, hyperglycaemia, and retarded growth [2].

In the pediatric age group, Cushing syndrome is very rare. It is estimated to occur in about 2–5 new cases per million individual per year [2]. The vast majority of these cases are iatrogenic. The most common cause of endogenous Cushing syndrome in preschool children is adrenal pathology such as hyperplasia, adenoma, or carcinoma [2]. Pituitary or ectopic causes are rare in this age group. On the other hand, iatrogenic (or exogenous) Cushing syndrome constitutes the vast majority of cases of Cushing syndrome among young children due to the high prevalence of diseases that require chronic use of corticosteroids such as bronchial asthma, pulmonary diseases, hematological diseases, renal diseases, or dermatological diseases [2, 3].

The diagnostic approach and differential diagnosis as well as the treatment of Cushing syndrome in general and iatrogenic Cushing syndrome, in particular, remain highly challenging in clinical endocrinology. Midnight salivary cortisol test, urine free cortisol (UFC) and low-dose dexamethasone suppression test (LDDST) have been shown effective as a primary screening for endogenous Cushing syndrome, particularly of the endogenous type, with the confirmation by urine free cortisol (UFC), low-dose dexamethasone suppression test (LDDST), and dexamethasone/corticotrophin-releasing hormone (DST-CRH) tests, a definitive criteria or a highly sensitive/specific diagnostic test for iatrogenic Cushing syndrome is still lacking in the literature [4].

However, one can diagnose Cushing syndrome of the exogenous type based on the clinical presentation of the classic signs of Cushing syndrome with the confirmation of a decreased 8 : 00 am basal cortisol level [5, 6].

The chronic use of corticosteroids is considered a widely prescribed medication for a variety of chronic conditions. However, when it is used for a prolonged period, it can result in the presentation of the systemic effects characteristic of Cushing syndrome. Moreover, this particular group of patients is subjected to develop tertiary adrenal insufficiency following the decrease or the elimination of corticosteroids. The mechanism through which this can occur is through the negative feedback loop on the hypothalamic-pituitary-adrenal (HPA) axis, which involves mainly corticotropin hormone (CRH) and adrenocorticotropic hormone (ACTH) release. Therefore, the reactivation of CRH is a necessity for the successful reactivation of the HPA axis after the elimination of chronic corticosteroid treatment [7].

Iatrogenic Cushing syndrome is common with the excessive use of oral and parenteral corticosteroids but rare with topical preparations [8]. In this article, we present an infant with iatrogenic Cushing syndrome after a two-month use of topical steroids.

## 2. Case Report

A three-and-a-half-month-old girl with an unremarkable past medical and developmental history presented to the emergency department (ER) with face puffing and generalized edema. Her mother stated that the girl's condition started insidiously two months before the presentation and progressed over time. There were no other associated symptoms and no history of recent trauma or insect bite. The mother reported that she has been using topical corticosteroid cream (clobetasol) for the past two months for the treatment of napkin dermatitis in a dose of five to eight times a day. The rash was subsided in two to three days and then flared up afterward. In conclusion, she was using a topical steroid cream on a continuous basis for two months.

Reviewing the infant's prenatal, developmental, past, and family history revealed that she was born full term to nonconsanguineous parents via caesarian delivery due to breech presentation. She was not admitted to a neonatal intensive care unit (NICU) after delivery and was discharged in good condition. She has been given exclusive breastfeeding every one to two hours, and she received all the required vaccinations at their proper times. She has one healthy sibling and negative family history of any relevant condition.

**Figure 1:**
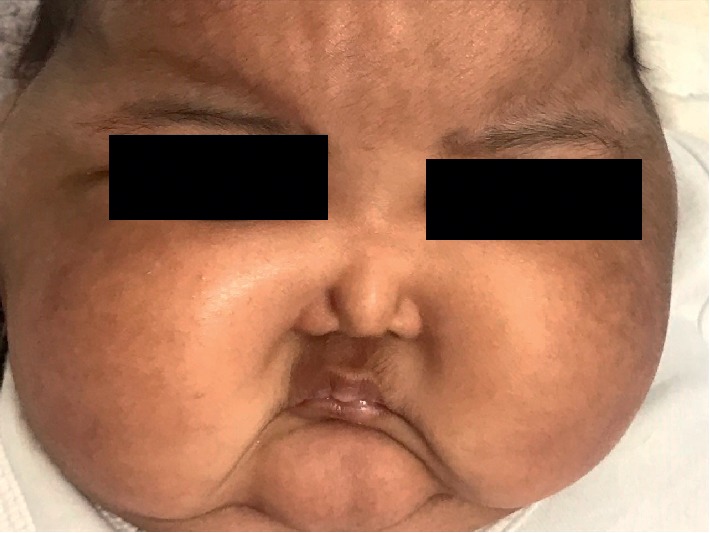
The patient's face showing facial puffiness and moon-face appearance.

**Figure 2:**
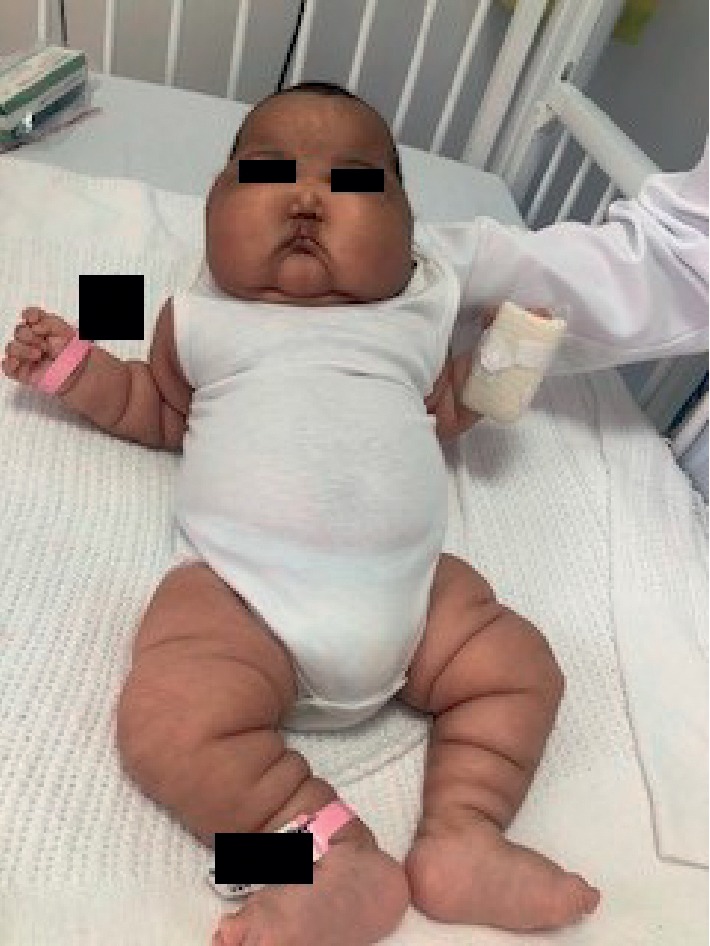
The patient's body showing generalized edema.

**Figure 3:**
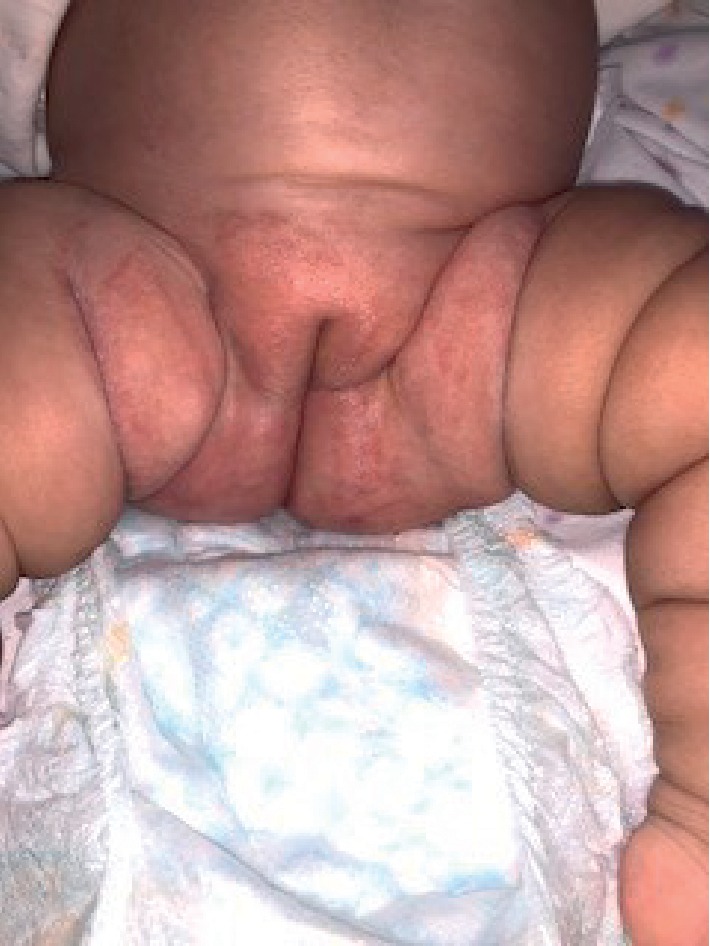
The patient's genitalia showing the napkin rash.

The patient's physical examination revealed facial puffiness (Figure 1) and generalized body edema (Figure 2). There were no abdominal stria, and the cutaneous examination revealed nothing but the napkin rash (Figure 3). The infant was vitally stable with no dysmorphic features and no skeletal deformities. Her growth parameters were within normal limits, and her systemic examination was unremarkable.

Upon investigation, the adrenocorticotropic hormone (ACTH) level was very low, 0.7 pg/mL, and the serum cortisol level was 17 *μ*g/dl. The abdominal ultrasonographic study showed normal adrenal glands.

Physiological doses of hydrocortisone (12 mg/m^2^) were administered to the patient for 6 weeks, and then 25% of the dose was tapered weekly. On the fifth week, ACTH stimulation was performed, where ACTH and cortisol levels were 5.7 pg/mL and 28 *μ*g/dl, respectively. Hydrocortisone was stopped while ACTH and cortisol levels were normalized. Clinically, facial puffiness and edema improved significantly over time.

## 3. Discussion

Under normal physiological conditions, the pituitary gland secretes ACTH that stimulates the adrenal glands to secrete cortisol. When corticosteroids are exogenously administered, suppression of this hypothalamic-pituitary-adrenal (HPA) axis occurs [3]. Though iatrogenic Cushing syndrome is common with prolonged administration of oral or parenteral steroids, some cases were reported to develop such condition upon prolonged use of topical steroids [9–12]. Few of these cases developed severe immunosuppression and fatal secondary infections [13].

The infant presented in this article developed iatrogenic Cushing syndrome after inappropriate and prolonged use of a highly potent topical steroid, i.e., clobetasol propionate for napkin dermatitis [14]. The mother used high doses of corticosteroid for two months consistently causing clobetasol to be absorbed through the skin, reaching the systemic circulation and resulting in the development of iatrogenic Cushing syndrome [15]. The clinical presentation of the child was typical for a case of Cushing syndrome including the signs of moon face, generalized edema, and weight gain, while her laboratory profile confirmed the diagnosis. ACTH was suppressed by the exogenously administered steroids, but cortisol levels were still within normal limits. Fortunately, our case was not complicated by the life-threatening adrenal insufficiency. Lately, the risk of developing life-threatening adrenal insufficiency has been evident in patients being administered almost any form of glucocorticoids, including topical creams. It has been known that glucocorticoid creams can lead to adrenal insufficiency even within the prescribed doses [16].

Unluckily, most of the symptoms of iatrogenic adrenal insufficiency are not specific and therefore are difficult to differentiate from other underlying disease pathology. Consequently, many clinicians and healthcare practitioners might not recognize such conditions and forget to include it in their differentials. Therefore, it is advisable to always keep iatrogenic adrenal insufficiency in mind in patients treated with all forms of steroids and presenting with iatrogenic Cushing syndrome [16].

The girl's height, blood pressure, skin, immune system, and blood glucose levels were not affected. Similar to our case, Siklar et al. [15], Sahip et al. [11], Güven et al. [13], and Tempark et al. [12] reported similar cases of iatrogenic Cushing syndrome after prolonged use of clobetasol for the treatment of napkin dermatitis. However, our case represents an early diagnosed case, where only facial puffiness and generalized edema developed with the suppression of ACTH level. The cases reported in the literature are rather advanced cases where the full-blown picture of the Cushing syndrome occurred.

In our case, clobetasol is not the topical steroid of choice for the management of the napkin rash. As shown in Figure 3, the rash is extensive, and the skin was inflamed and damaged. This allows more permeation and penetration of the topical medication. Thus, it was better to choose a low potency topical steroid, e.g., hydrocortisone than to choose a very potent agent such as clobetasol [17]. Furthermore, screening methods such as midnight salivary cortisol could prove to be helpful in early detection of potential iatrogenic Cushing syndrome while using topical steroids [18]. Management of iatrogenic Cushing syndrome includes the stoppage of the causative medication, administration of exogenous physiological doses of steroids, and tapering them slowly over time [2].

The presented case, along with the previously published cases in the literature, should warrant the importance of adequate health education of the mothers and parents about the appropriate instructions of using any topical steroid preparation. Physicians should instruct them to apply thin layers of the topical preparation and not to exceed the daily dose of treatment, while these preparations should only be for the short-term use.
